# VirJenDB: a FAIR (meta)data and bioinformatics platform for all viruses

**DOI:** 10.1093/nar/gkaf1224

**Published:** 2025-12-17

**Authors:** Shahram Saghaei, Malte Siemers, Kilian L Ossetek, Stephan Richter, Robert A Edwards, Simon Roux, Andrzej Zielezinski, Bas E Dutilh, Manja Marz, Noriko A Cassman

**Affiliations:** RNA Bioinformatics and High-Throughput Analysis, Faculty of Computer Science and Mathematics, Friedrich Schiller University Jena, Jena 07743, Germany; Institute of Biodiversity, Ecology, and Evolution, Faculty of Biological Sciences, Cluster of Excellence Balance of the Microverse, Friedrich Schiller University Jena, Jena 07745, Germany; RNA Bioinformatics and High-Throughput Analysis, Faculty of Computer Science and Mathematics, Friedrich Schiller University Jena, Jena 07743, Germany; RNA Bioinformatics and High-Throughput Analysis, Faculty of Computer Science and Mathematics, Friedrich Schiller University Jena, Jena 07743, Germany; Flinders Accelerator for Microbiome Exploration, College of Science and Engineering, Flinders University, Adelaide 5001, Australia; U.S. Department of Energy Joint Genome Institute, Lawrence Berkeley National Laboratory, Berkeley, CA 94720, United States; Institute of Molecular Biology and Biotechnology, Faculty of Biology, Adam Mickiewicz University, Poznan 61-712, Poland; Institute of Biodiversity, Ecology, and Evolution, Faculty of Biological Sciences, Cluster of Excellence Balance of the Microverse, Friedrich Schiller University Jena, Jena 07745, Germany; Theoretical Biology and Bioinformatics, Science4Life, Utrecht University, Utrecht 3584 CH, The Netherlands; RNA Bioinformatics and High-Throughput Analysis, Faculty of Computer Science and Mathematics, Friedrich Schiller University Jena, Jena 07743, Germany; European Virus Bioinformatics Center, Jena 07743, Germany; Leibniz Institute on Aging, Fritz Lipmann Institute eV, Jena 07745, Germany; Bioinformatics Core Facility, Friedrich Schiller University Jena, Jena 07743, Germany; RNA Bioinformatics and High-Throughput Analysis, Faculty of Computer Science and Mathematics, Friedrich Schiller University Jena, Jena 07743, Germany; European Virus Bioinformatics Center, Jena 07743, Germany

## Abstract

High-throughput sequencing has generated an unprecedented volume of data. However, researcher-submitted data in repositories requires extensive curation and quality control for reuse. These tasks are hindered by the multiplicity of repositories, the sheer volume of the data, and the complexity of virus (meta)data curation. To address these challenges, VirJenDB offers a user-friendly platform to facilitate versioned, community-driven curation, and ontology development. Virus sequences were ingested from 16 sources, including ~200 fields of metadata or standards, covering taxonomy, sample, and host information. Up to 85 metadata fields have undergone at least one round of curation, and are linked to 15.4 million virus sequences, with 88 % from those infecting eukaryotes and the remaining infecting prokaryotes. Subsets were created, including a novel collection of 0.91 million viral operational taxonomic unit (vOTU) sequences across all viruses, while keeping the original sequences from each vOTU to facilitate downstream analyses, e.g. sequence variation. The VirJenDB web portal (https://www.virjendb.org) provides HTTPS and Application Programming Interface (API) access to the sequence datasets and metadata, offering a search engine, filtering, download, visualizations, and documentation. VirJenDB aims to connect the phage and eukaryotic virus research communities by supporting webtool integration, meta-analyses, and metadata schema extensions.

## Graphical Abstract

**Graphical Abstract:**
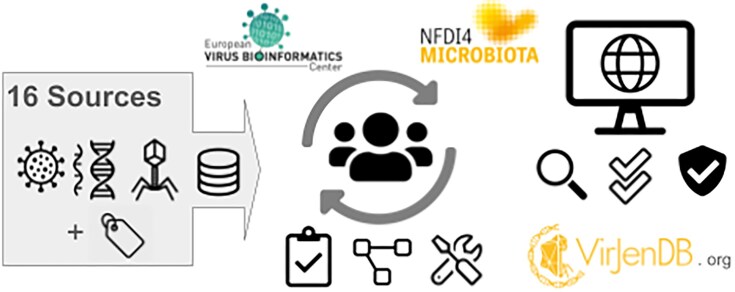

## Introduction

The application of high-throughput sequencing has led to an unprecedented accumulation of virus sequence data, including from genomic and metagenomic data. Virus sequences are stored in primary repositories [1–4], which together hold tens of millions of virus sequences and metadata with varied degrees of annotation. User-submitted data and downstream data layers are a highly valuable public resource [5, 6], but can contain errors in user inputs during the uploading process, which remain challenging to address or repair [7]. To enable the transparent reuse of user-submitted data, additional curation and quality control, together with their provenance, are required [8]. Curation efforts may be provided by the primary repositories themselves or by secondary databases, including well-known databases, portals, and data dashboards [9–12]. However, the volume of data poses a challenge to these efforts; moreover, the curation steps are rarely made public in detail, e.g. are not assigned unique identifiers nor versioned, which is needed for alignment with the Findable, Accessible, Interoperable, and Reproducible (FAIR) principles and in creating Transparent, Responsible, User-focused, Sustainable, and Technologically relevant (TRUST) digital resources for virus data and metadata [8, 13]. Further, the lack of virus-specific ontologies and data models may lead to redundant curation efforts. Given the rapidly expanding view of the virosphere, the scope and complexity of viruses, and the diversity of the virology research community, an accessible and well-structured virus data and metadata repository is both needed and timely.

As viruses are obligate genetic parasites, the host is one of the most important metadata fields. However, current virus–host resources often lack support for associating multiple hosts to a virus, and for annotating host associations with source information (e.g. experimental evidence or computational prediction) and confidence scores [11, 14, 15]. This problem is further complicated by discrepancies between virus taxonomy resources [16, 17] and by inconsistent use of host taxonomic classification systems, which range from strain-level specificity to broad phylogenetic groupings and rely on distinct classification schemes such as SILVA, NCBI, or GTDB [16, 18, 19]. Moreover, virus sequence resources are often divided into phage and eukaryotic viruses [20, 21, 38], creating barriers for researchers aiming to interpret viral metagenomics sequencing data, synthesize findings across studies, or conduct meta-analyses of all viruses. Finally, virus taxonomic heterogeneity not only complicates efforts to identify patterns in host range and specificity but also limits the development of predictive models for virus and host evolution, phage therapy applications, and studies of viral impacts on the microbiome or microbial ecology.

Here, we present VirJenDB, an integrative virus database borne from the needs of the European Virus Bioinformatics Center (EVBC) [22, 24] community, developed within the German National Research Data Infrastructure for Microbiota (NFDI4Microbiota) consortium [25] and based in Jena, Germany. The release includes several comprehensive datasets of all viruses accessible through a user-friendly, freely available web portal at https://www.virjendb.org, which offers advanced search, download, and visualization functions. Importantly, VirJenDB supports community-driven development of metadata schema extensions through the GitHub repository (https://github.com/virjenDB), enabling enrichment and refinement of virus metadata and facilitating improved sequence deposition to INSDC primary repositories. By supporting ecological, meta-analysis and artificial intelligence (AI)-driven research on all viruses, VirJenDB serves as a unifying infrastructure bridging the research communities studying viruses of eukaryotes and prokaryotes in Germany and internationally, in alignment with FAIR and Open Science principles.

## Materials and methods

### Harvesting and integration

To create the VirJenDB dataset, virus sequences and metadata were ingested, merged, harmonized, and curated from publicly available primary and secondary sources. The 11 sources of metadata and standards are listed in Table 1. Sequences were ingested from the five sources listed in Table 2. Each virus record was assigned a unique VirJenDB Accession as identifier. Identifiers from the source databases were kept where available. The metadata information was used to merge sequence data from different sources, with the INSDC Accession ID or BV-BRC ID as the primary key.

**Table 1.** Sources of metadata and standards used for mapping and merging to obtain the VirJenDB v1.0 metadata schema, including the fields accessible on the web portal

Metadata or standard source	URL	Version and/or access date	Number of fields	Citation
NCBI Virus 1.0 schema	https://www.ncbi.nlm.nih.gov/biosample/docs/packages/Virus.1.0	1.0	26	[11]
BV-BRC results table	https://www.bv-brc.org/view/Taxonomy/10239#view_tab=genomes	19 February 2025	33	[12]
GSC Miuvig	https://genomicsstandardsconsortium.github.io/mixs/0010012	–	10	[27]
GSC MigsVi	https://genomicsstandardsconsortium.github.io/mixs/0010005	–	8	–
ENA Influenza virus reporting standard checklist	https://www.ebi.ac.uk/ena/browser/view/ERC000032	–	12	[4]
ENA virus pathogen reporting standard checklist	https://www.ebi.ac.uk/ena/browser/view/ERC000033	–	12	[4]
RKI	https://github.com/robert-koch-institut/SARS-CoV-2-Sequenzdaten_aus_Deutschland		5	[28]
ICTV Virus Metadata Resource and Master Species List	https://ictv.global/vmr, https://ictv.global/msl	VMR_MSL40.v1MSL40.v1	14	[17]
PubMed	https://pubmed.ncbi.nlm.nih.gov	12 November 2023	1	–
NCBI Taxonomy	https://www.ncbi.nlm.nih.gov/taxonomy	12 November 2023	15	[16]
ViralZone	https://viralzone.expasy.org		2	[29]

**Table 2.** Sources of virus sequences ingested to construct the VirJenDB v1.0 full dataset

Data source	URL	Version and/or access date	DOI	Number of sequences	Citation
BV-BRC	https://www.bv-brc.org	v3.32.13a	10.1093/nar/gkac1003	BV-BRC seqs: 878 067(GenBank/RefSeq: 11 341 775)	[12]
NCBI Virus	https://www.ncbi.nlm.nih.gov/labs/virus/vssi/	12 November 2024		13 053 765	[11]
PhiSpy catalog	https://doi.org/10.25451/flinders.22317059.v1	11 April 2023	10.25451/flinders.22317059.v1	1 217 941	[30]
IMG/VR	https://img.jgi.doe.gov/vr	V4.0	10.1093/nar/gkac1037	198 525	[9]
Phage and Host Daily (NCBI GenBank, NCBI RefSeq, NCBI Taxonomy, GTDB)	http://www.phdaily.info	17 February 2025	10.3389/fmicb.2022.946070	3204 (GenBank/RefSeq: 24 204)	[15]

The input source fields were mapped into a metadata spreadsheet, the VirJenDB metadata schema v1.0 (Supplementary Table S1). These included metadata field identifiers, names, descriptions, types, source db, and other meta information. Fields were classified as public or internal, corresponding to whether or not these would be integrated into the full functionality of the website, which can be switched as needed. Light curation was carried out during data ingestion to improve consistency and interoperability, including reformatting dates to a standardized ISO 8601 format, splitting multi-value fields into discrete entries, and correcting common typographical errors in metadata fields. More extensive curation was carried out on the host-related fields as detailed below.

### Quality control and curation

Fields were added to the VirJenDB metadata schema by calculations or workflows carried out by the VirJenDB, noted as containing derived information (20 derived fields). Several of these are flags with BOOLEAN values indicating presence in a set (GenBank Sequence, RefSeq Sequence, Unique Representative, Cluster Representative, and ViralZone Page). To address inconsistencies in the phage host taxonomic classification across datasets, we standardized all host information to the GTDB v226 taxonomic framework. The NCBI taxonomic assignments were converted to GTDB taxonomy using GTDB metadata files. For each NCBI species, we mapped to the corresponding GTDB species by identifying the mode of the distribution across all associated GTDB species assignments, ensuring that the most frequently represented GTDB classification was selected for each NCBI taxon. To include viruses of prokaryotes with confident host assignments, we compiled data from three major repositories. First, we downloaded a catalog of 5.05 million predicted prophage sequences by PhiSpy version 4.2.21 [30, 31]. Second, we obtained the high-confidence subset of the IMG/VR v4.0 database, comprising 5.58 million sequences [9]. Finally, we incorporated 27 408 sequences from the PHD [15] dataset, which included phages with high-quality host information mined from seven sources: NCBI Virus [11], Virus-Host DB [14], MVP [32], RefSeq [10], GenBank [1], UniProt [33], and IntAct [34].

The phage datasets were filtered to retain only records with host information available at the species level. For PhiSpy prophage predictions, host information was obtained by mapping the prophage host genome identifiers with the NCBI TaxIds from the BV-BRC input dataset. From IMG/VR v4.0, we extracted phage sequences with host information available in the GTDB v207 taxonomic scheme, specifically selecting entries tagged as “isolation host” from the associated metadata. The PHD dataset provided host information in both NCBI and GTDB v220 taxonomic schemes, which we incorporated directly into our dataset. This filtering step resulted in 1.21 million sequences obtained from the PhiSpy predictions, 199 thousand sequences from IMG/VR4 and 27 000 sequences from the PHD dataset, of which 24 000 had overlap with the VirJenDB sequences from GenBank/RefSeq. These 1.47 million phage sequences were merged with associated metadata, including “Host NCBI TaxID” and “Host NCBI Species Name,” into the VirJenDB dataset (hereafter referred to as the full dataset).

### Summary plots construction

To visualize the content of the full dataset and metadata, plots were created from the number of virus records within the fields: NCBI Family, Host Species, Molecule Type, Submitter Country, GC Content, and Sequence Length. As an overview, host TaxID presence in the GTDB was used to group records as viruses with either a prokaryotic or eukaryotic host.

### Sequence subsets: dereplication, clustering, and selection of representatives

To supplement the full dataset, we created subsets for various user bases. By deduplicating and clustering the sequences, we aimed to capture the known diversity of the virus sequence space represented in the full dataset in a smaller file. Sequence dereplication was performed using a multi-step approach here called the Vclust vOTU workflow (Supplementary Fig. S1). First, exact duplicate sequences were removed through hash-based dereplication, resulting in the deduplicated dataset, which we provided. Due to the large volume of sequences surpassing the capacity of Vclust, the deduplicated dataset file was split into subfiles of ~1 million sequences and each subfile was clustered at 95% similarity using linclust from mmseq2 v14.7e284 [35]. The linclust cluster subfiles were then clustered at 95% average nucleotide identity (ANI) over 85% of the query sequence length using Vclust 1.30 [36] with the Leiden algorithm to obtain the Vclust step 1 clusters. Last, the Vclust clusters were merged into one file, and once again de-replicated using Vclust at 95% ANI over 85% query length with the Leiden algorithm to obtain the final step 2 vOTU clusters (hereafter referred to as the vOTUs). After manual inspection of the results, we identified an abnormally long SARS-CoV-2 sequence of ~10 million nucleotides in length which had been selected as a vOTU representative. We manually selected the second-longest sequence within this vOTU to be the representative instead. Note that we kept the linking of which vOTU every input sequence was clustered into, and added this as a metadata field “Cluster Reference” to the full dataset. Additional derived metadata fields added from the Vclust vOTU workflow were: “Cluster Representative,” “Unique Representative,” and “Unique Reference.” Further, to evaluate the quality of the vOTU dataset, the CheckV (v1.0.1) [26] end-to-end workflow against the v1.5 CheckV database was run on every vOTU representative.

### UMAP projection of the connected vOTU components

To visualize the similar vOTUs, pairwise ANI distances >0.7 over 85% of the sequence length were computed using Vclust. The ANI distances were used to construct a graph, from which connected components were identified. For each connected component, the pairwise distance matrix of sequences within that component was used to compute Uniform Manifold Approximation and Projection (UMAP) embeddings of the similar vOTUs. For visualization purposes, the 100 largest connected components were selected. The UMAP coordinates of each component were positioned within squares scaled relative to the cube root of the component size, and a guillotine bin packing algorithm was applied to arrange these squares adjacently in the final visualization layout. To explore the connected component visualization, the public metadata fields of the VirJenDB were used to color these by NCBI virus species.

### The vOTU comparison to external databases

To contextualize the scale and scope of the VirJenDB, we compared our vOTU dataset to three established nonredundant viral sequence databases (INPHARED, PhageScope, RVDB). We downloaded the INPHARED: 14 April 2025, PhageScope: v1.3, downloaded 12 September 2025, RVDB: C-RVDB v30.0 datasets which contained 34 076, 873 718, and 1.2 M sequences, respectively. To be able to compare sequence diversity between these datasets, we clustered the sequences provided by the respective source with Vclust at 95% ANI over 85% length with the Leiden algorithm and counted the number of resulting clusters.

### Backend implementation

The central service of the VirJenDB is a web server written in the Python programming language (ver 3.10, https://www.python.org). It utilizes Python’s FastAPI framework to handle requests from the frontend and Application Programming Interface (API) users. This Python service interacts with an instance of ElasticSearch (ver 9.0.4, https://www.elastic.co), handling queries corresponding to the API requests.

We chose Elasticsearch as it is an open-source tool providing fast and powerful means to query the dataset by indexing fields and returning search results in JSON format. Also, it supports structured yet flexible metadata storage, facilitating future extension and scalability. Retrieved data are processed by the backend and returned to the client. Public backend services are accessed through an NGINX proxy, which routes requests and responses appropriately. Supplementary Figure S2 presents the database network architecture, detailing the flow of user requests and system responses. Programmatic requests are handled contextually, either as server-side queries or as files retrieved from Aruna object storage.

The database service and the server processes are managed as an OpenStack project on the de.NBI cloud (https://www.denbi.de). Sequence data are stored on the data orchestration engine Aruna (https://aruna-engine.org) as well as the Uni Jena servers and accessed via the VirJenDB portal from these sources. Development takes place on several cloud-hosted instances, while source code is managed using the GIT version control system and stored in the VirJenDB GitHub repository (Supplementary Fig. S3).

For security reasons, all interactions between clients (Frontend and API users) and backend services utilize the HTTP protocol via secure socket layer (HTTPS). As a measure to enable system recovery after potential failure, we implemented a backup routine, creating an Elasticsearch snapshot of the dataset (stored in a separate de.NBI cloud instance) and a MySQL dump (Version 8.4, https://www.mysql.com) downloaded to local storage with each VirJenDB release.

### Frontend implementation

The VirJenDB dataset is accessible via a dedicated web interface built using the React JavaScript framework (Version 18.2.0, https://www.react.dev). We utilized the Shadcn/ui component library to ensure consistent user interface behavior and accessibility. The development of the web interface is driven by a user-centered approach. Since the beginning of the development, our team has been in exchange with users at conferences and in workshops, either in personal interviews or via surveys, to improve webpage navigation and usability and investigate the community’s needs. We additionally run intensive in-person bug tests and interviews with a variety of users from different research fields to enrich our understanding of their needs. In order to further improve the user experience we use privacy-protected user tracking using Matomo (https://matomo.org).

## Results

### Comprehensive metadata integration

VirJenDB has integrated 201 source fields from 16 sources of virus sequences, metadata, and standards. In addition, we derived 15 fields, resulting in a unified set of 216 fields, of which we present 86 public fields (including 10 derived fields) available through the current release. The VirJenDBv1.0 metadata schema is available on the website documentation, the VirJenDB Metadata GitHub repository (https://github.com/VirJenDB/virjendb_metadata) and in an abbreviated format in Supplementary Table S1. Of all public fields, 9 fields were empty while 77 contained at least one value, with data completeness ranging from 0.1% to 100% (Supplementary Fig. S4). The VirJenDB fields comprise two broad groupings, including Group 1: General (Organizational, Sample, Source, Host, Analysis, Taxonomy) and Group 2: Technical (Identifiers, Collection, Flags, Host Taxonomy, Workflows, Virus Info, Submission, Sequence, Host Info, Clinical, Source, Virus Taxonomy). The metadata field names, descriptions, types, example values, and, where available, controlled vocabulary are accessible at the documentation page of the database (https://doc.virjendb.org) and in Supplementary Table S1. In addition, users can contribute new metadata fields, as further explained below in the section “User-contributed metadata.”

### Sequence overview: virus taxonomic diversity and host skew

**Figure 1.**
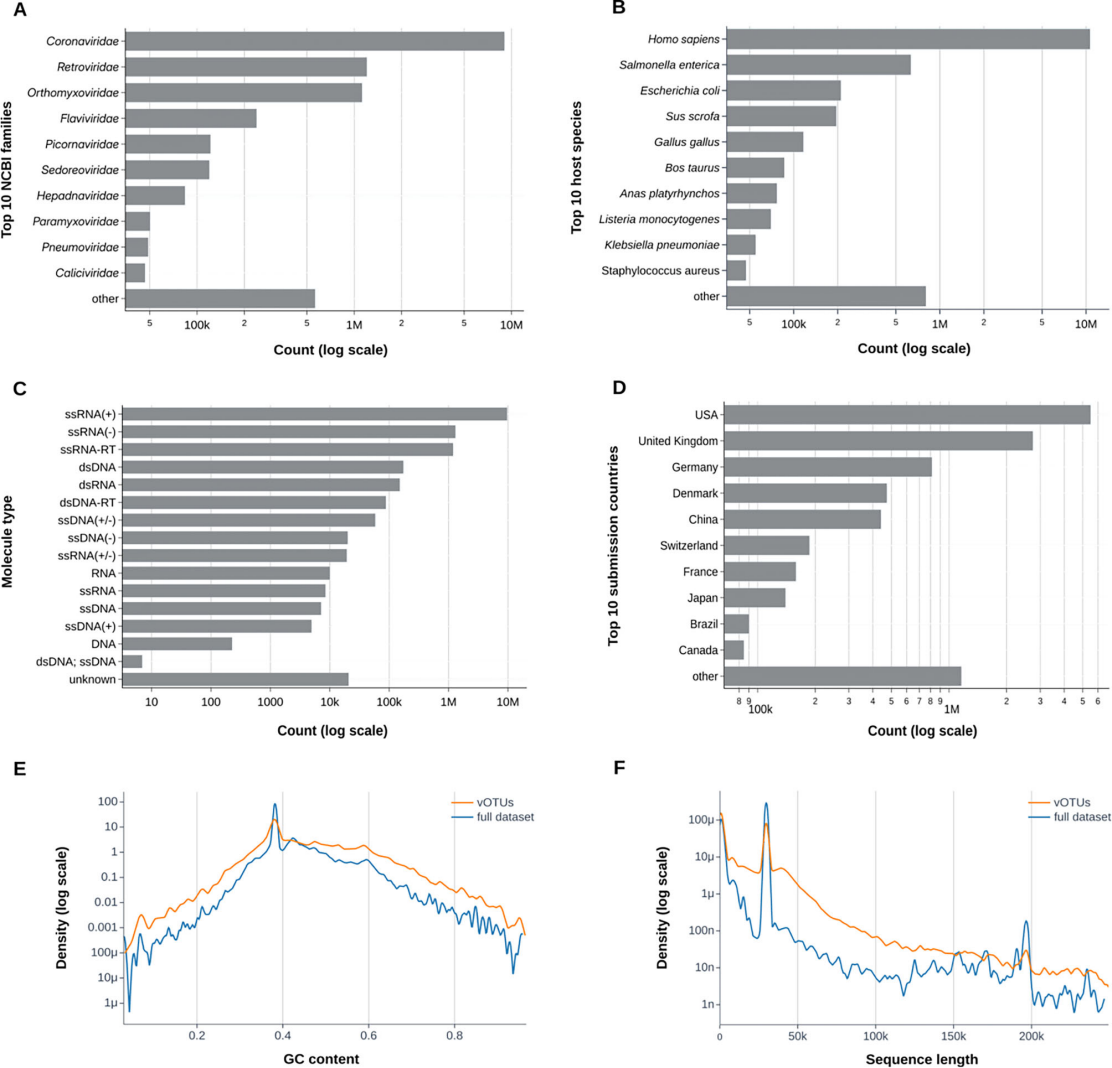
Summary plots of the VirJenDB content. Barplots of the (**A**) top 10 NCBI virus taxonomic families, (**B**) top 10 annotated host species, (**C**) molecule types according to the available data in NCBI GenBank and RefSeq, which will be unified/hand-corrected in future releases, (**D**) top 10 submission countries. Logarithmic densities of (**E**) the GC content of the sequences and (**F**) the sequence length of sequences shorter than 250k nucleotides. Densities are shown in blue for the full dataset and in orange for the vOTUs.

To date, VirJenDB contains 15 351 501 viral sequences, of which 3 293 428 were annotated as complete genomes in NCBI Virus. In NCBI Virus, the sequence completeness is determined in most cases by the submitter annotations to GenBank or other INSDC databases. The full dataset covers 267 virus families, 2763 genera, and 52 815 species. Overall, there was a pronounced skew toward viruses of eukaryotes, which comprised of 11.5 million sequences (88%) compared to 1.4 million sequences (11%) from viruses of prokaryotes. The eukaryotic virus families *Coronaviridae, Retroviridae*, and *Orthomyxoviridae* were the Top 3 most represented with 9.0 million, 1.3 million, and 1.2 million records (Fig. 1A); the remaining virus families in the top 10 were *Flavivi-, Picorna-. Sedoreo-, Hepadna-, Paramyxo-, Pneumo-*, and *Caliciviridae*.

The most represented host species, *Homo sapiens*, dominated the full dataset with 10.7 million sequences, representing over 93% of all eukaryote-associated virus sequences (Fig. 1B). Other eukaryotic host species in the top 10 were *Sus scrofa, Gallus gallus, Bos taurus*, and *Ana platyrhynchos*. Prokaryotic host species in the top 10 included *Listeria monocytogenes, Klebsiella pneumoniae*, and *Staphylococcus aureus*. The top second and third hosts were prokaryotic and consisted of *Salmonella enterica* accounting for 630 000 sequences (45% of prokaryote-associated viruses) and *Escherichia coli* contributing 210 000 sequences (15%, Fig. 1B).

Virus genomes may be encoded on different molecule types, and VirJenDB includes 12.6 million RNA viruses and 380 000 DNA viruses (Fig. 1C), reflective of the distribution of virus families. Most viral sequences were submitted to repositories from the USA, followed by the UK and Germany (5.5 million, 2.8 million, and 860 000; Fig. 1D). The GC content of most of the sequences ranged from 30% to 60% (Fig. 1E, blue line) while sequence lengths were predominantly shorter than 50k nucleotides (Fig. 1F, blue line).

### Overview of the vOTUs

Through systematic dereplication, a deduplicated subset was obtained from the full dataset containing 12 835 810 sequences. Further, through clustering at the MIUViG species-level cutoff 95% ANI over 85% genome length, we obtained a nonredundant collection of 911 648 representative genomes, representing approximately species-rank viral operational taxonomic units (vOTUs, Supplementary Fig. S1A, 26). Of the vOTUs, 630 000 turned out to be singletons (Supplementary Fig. S1B). The largest vOTU cluster comprised of 4.5 million sequences and could be identified as SARS-CoV-2. When we used CheckV to predict the genome completeness of the vOTU sequences, we surprisingly found that only 4251 sequences were predicted as complete, 281 706 were high-quality, 58 570 were medium quality, and 322 771 were low quality, with the remainder not determined (244,178).

The GC content of most of the vOTUs ranged from 30% to 60% (Fig. 1E, orange line) while sequence lengths were predominantly shorter than 50k nucleotides although higher than the full dataset, reflecting the choice of vOTU representative by longer sequence length (Fig. 1F, orange line). Compared to the full dataset, the distributions of GC content and sequence length were both spread out more evenly (Fig. 1E and F, orange lines), highlighting the reduction of the bias toward single viruses like SARS-CoV-2 and Influenza A in the vOTU dataset.

### Comparison of the vOTUs with external databases

To contextualize the scale and scope of VirJenDB, we compared our dataset to three established nonredundant viral sequence databases. Inphared, which focuses exclusively on bacteriophages, contains 12 770 representative sequences clustered at 95% similarity [20]. RVDB provides a broader scope with 1.2 million sequences covering both prokaryotic and eukaryotic viruses, clustered at a more stringent 98% similarity threshold [21]. PhageScope (v1.3) uses some of the same data sources as VirJenDB, including 7169 sequences from NCBI Genbank/RefSeq and 177 361 from IMGVR, with additional sources of phage-specific catalog databases up to 873 718 sequences [23]. To be able to directly compare the diversity of sequences in RVDB, we clustered them with Vclust at 95% ANI over 85% length. This procedure led to 12 495 clusters in the INPHARED dataset, 358 108 clusters derived from PhageScope, and 503 340 clusters in the RVDB dataset compared to VirJenDB’s 911 648 (Supplementary Fig. S6).

### Visualization of the connected vOTUs

**Figure 2.**
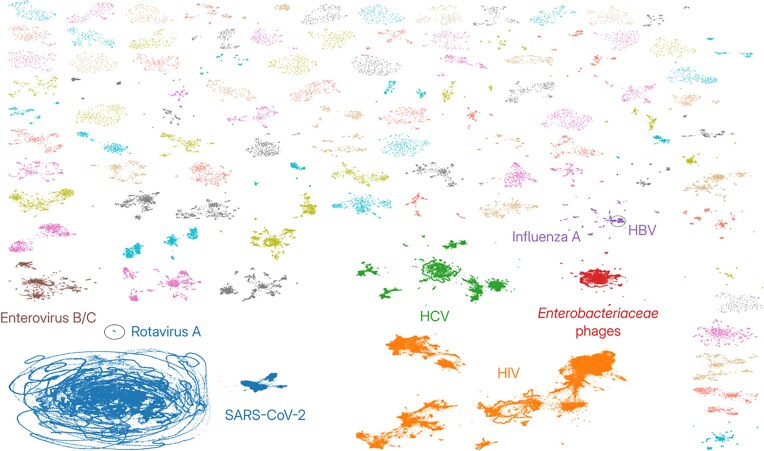
UMAP visualization of the species-level vOTU representative genomes connected by pairwise ANI values ≥ 0.7 as calculated by Vclust (see Materials and methods). The 100 largest connected components (total: 436 979 representatives) are shown in different colors. The six largest connected components are labeled, representing severe acute respiratory syndrome coronavirus 2 and Rotavirus A (SARS-CoV-2/Rotavirus A in dark blue), human immunodeficiency viruses (HIV in orange), hepatitis C virus (HCV in green), bacteriophages infecting *Enterobacteriaceae* bacteria (red), Influenza A virus and hepatitis b virus (Influenza A/HBV in purple), and Enteroviruses B and C in brown. Note that the distance between connected components is meaningless.

To visualize the connected vOTUs and get an overall summary of the virus sequence space contained in VirJenDB, we performed a secondary clustering of the vOTU representatives using more relaxed criteria (70% ANI over 85% sequence length) resulting in 17 342 connected components with more than one member and 505 481 singletons. When the pairwise ANI distances were projected using UMAP, this revealed the two largest connected components that each contained ~175 000 vOTU representatives, including SARS-CoV-2 and HIV sequences. Interestingly, the SARS-CoV-2 component featured a large “hairball” with 140 000 vOTUs and a more condensed part to the right containing 30 000 vOTUs. The third- and fourth-largest connected components included sequences of hepatitis C viruses and *Enterobacteriaceae* bacteriophages, respectively. The fifth and sixth largest components were mainly attributed to Influenza A and Enteroviruses B and C. Interestingly, the largest SARS-CoV-2 connected component contained a small set of sequences labeled as Rotavirus A while the Influenza A component also included representatives of the hepatitis B virus (Fig. 2 and Supplementary Fig. S5). The latter case was explained by the presence of several synthetic expression vectors in our database which contain parts of both the Influenza A and Hepatitis B virus genomes.

### Web portal

**Figure 3.**
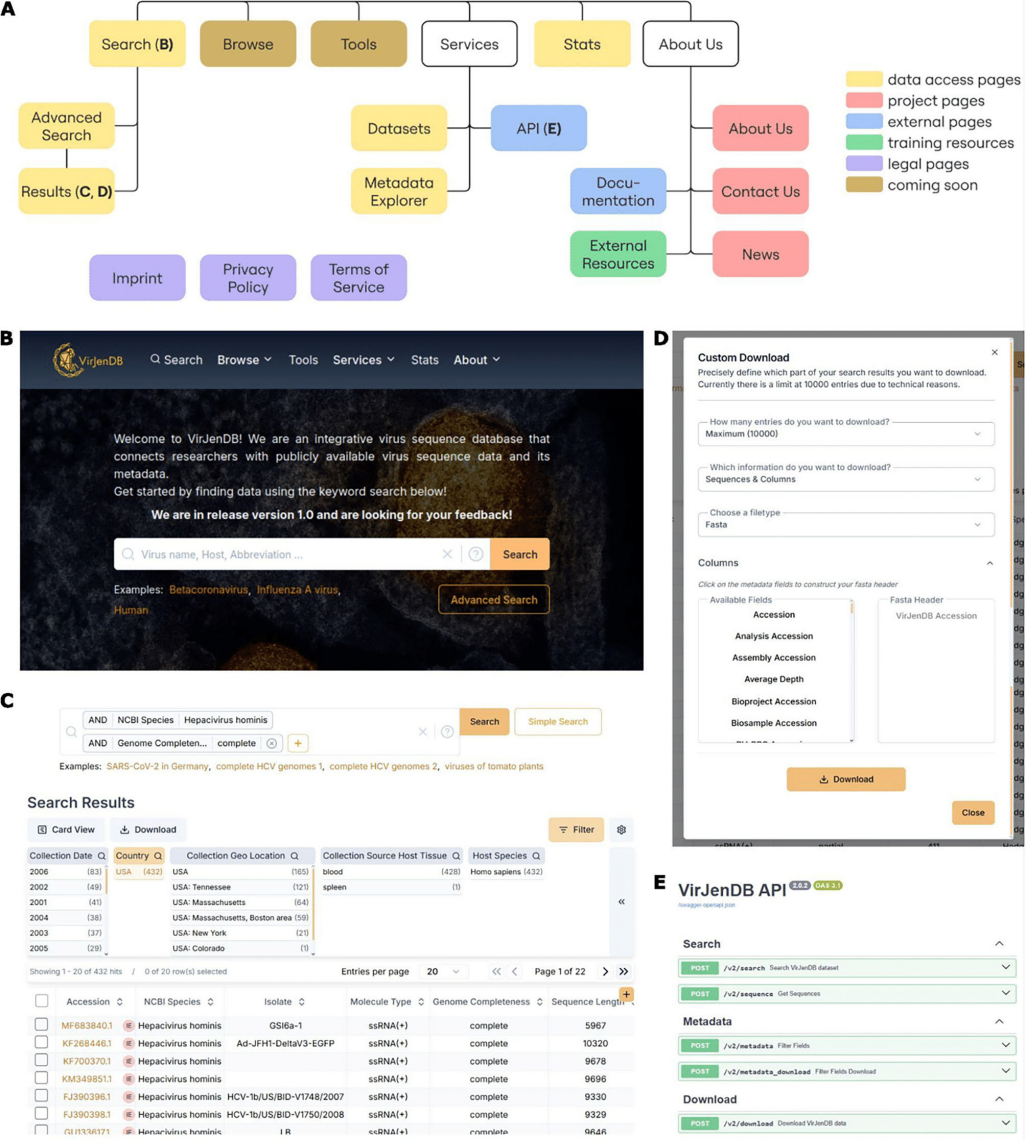
Overview of the VirJenDB website. (**A**) Menu structure of the VirJenDB web interface at https://virjendb.org. Each colored block describes a page. Users gain access to the full dataset (data access pages, yellow) through search, filter, display, and download features, further visualized in (**B–E**). Additional pages explain the VirJenDB project (red), help pages (blue), training information (green), and legals (purple). (B) Screenshot of the homepage, featuring the Simple Search box to query the full dataset. (C) Screenshot of the Search Results page, showing the features (from top to bottom) “Advanced Search,” “Filter” for two-step fine-grained queries, and the “Results Table,” which displays the query results. (**D**) Screenshot of the “Custom Download” pop-up feature on the Search Results page, which allows users to download their refined data subset in different file formats and with custom metadata. (E) Screenshot of the external SwaggerUI API page, providing access to the full dataset.

The web portal at https://virjendb.org serves as the central VirJenDB access point. Through several functionality pages, users have access to the full dataset of metadata and virus genome sequences via search, download, and visualization options (Fig. 3A). In addition, users can find links to our external help pages, the API SwaggerUI page, training resources, project information, and legal pages. For any inquiries about the functionality of the webpage or its contents, we provide an anonymous feedback form and database email address, both featured on our Contact Us page (https://virjendb.org/ContactUs).

### Search and results

We provide two query functions that rely on semantic search through the VirJenDB metadata to find matching records:

1. Simple Search on the front page serves as a low-barrier entry point (Fig. 3B). This allows users to query the database using a single search term, which is checked for matches in a defined list of 19 metadata fields: Sequence Name, ICTV Virus name, Abbreviation, Accession, Host Common Name, Organism Name, SRA Accession, BV-BRC Accession, Host Species, Host NCBI Tax ID, NCBI SpeciesTax ID, VirJenDB Accession, ICTV Species, NCBI Family, NCBI Genus, NCBI Species, Isolate, ICTV Host Group, Host AccessionSequence Name, ICTV Virus name, Abbreviation, Accession, Host Common Name, Organism Name, SRA Accession, BV-BRC Accession, Host Species, Host NCBI Tax ID, NCBI SpeciesTax ID, VirJenDB Accession, ICTV Species, NCBI Family, NCBI Genus, NCBI Species, Isolate, ICTV Host Group, and Host Accession.
2. Advanced Search (https://virjendb.org/AdvancedSearch) allows a more controlled and precise query. Provided search terms are only checked in defined metadata fields, allowing for high precision. Multiple search terms may be combined via logical operators (AND, OR, NOT).

To return to a certain search at a later point, the users’ last 20 searches are stored in their browser’s local storage together with a time stamp as well as the number of hits of the search (sequence entries). Thus, the searches are not stored by VirJenDB.

Search results comprise a subset of the full dataset that will be presented on the results page (https://virjendb.org/Results) (Fig. 3C). This page visualizes the entries found by the user’s search. Each entry is represented as a row (Table View) or a card (Card View). Clicking an entry provides the user with an overview of all available metadata and the sequence for that entry to the right of the screen. Additionally, the Table View (Fig. 3C) allows for more integrated investigation of the search results. All available metadata fields are available as columns and can be sorted by. Each row is selectable for fast access via download. Both, Card, and Table View feature pagination, increasing speed and visibility of the retrieved search results and precise filter options. For a selection of 22 fields we provide a list of unique and clickable values found within the search results. Applying the filter to these fields will limit the search results to the chosen value (Fig. 3C).

### Download of selected results and datasets page

Once the selection fits the user’s needs, they can access the selected records on the Results Table page using the Download button. Several data formats are offered to facilitate the integration of the downloaded data into the user’s research workflows, including FASTA with custom headers for sequence downloads, and TSV, CSV, JSON, and XML for selected metadata downloads (Fig. 3D). Currently, up to 10 000 records with all available metadata fields can be downloaded via the Result Table. Larger data files are provided for download on a separate Datasets page (https://www.virjendb.org/Datasets). We provide several commonly used, pre-selected datasets that can be downloaded directly on the datasets page or programmatically by using provided wget and curl commands. Accessible subsets include commonly requested queries, as well as VirJenDB-derived datasets, such as the representative genomes of the vOTU clusters.

### Taxonomy browser

In addition to the metadata search described above, the Taxonomy Browser (https://www.virjendb.org/Browse) page allows users to search the dataset by using the NCBI Taxonomy hierarchy [16]. Rank names are displayed on the left of the page in a tree-like structure starting at the highest rank: taxonomy ID 10239 (“Viruses”). To the left of every displayed rank name and taxa name there is a Plus button, which when clicked will expand the tree to show all highest order children of the clicked rank name. When clicked, the Minus button will collapse the tree again. This button is not visible if there are no children available for this rank name. On the right side of each displayed taxa name, a Search button (magnifying glass icon) is shown. When the Search icon is selected, the Results page with the searched taxa name will be loaded, utilizing the Advanced Search. This button is only available for 15 ranks featured in the metadata schema, therefore searching for taxa names such as “No Rank” or “Clade” are not currently supported. If available, the ViralZone icon is shown left of the Search button, linking to the external ViralZone page of the corresponding taxa.

On the right side of the page, there is a selection of featured virus species, displayed by ViralZone images, names and if available a ViralZone icon that links to the corresponding external ViralZone page. Clicking the respective image will search for the shown species by utilizing the Advanced Search and load the Results Table.

### Metadata explorer page

The VirJenDB Metadata Explorer feature allows for quick, structured access to all public metadata fields (https://virjendb.org/MetadataExplorer). Each metadata field is categorized using labels, or tags, which are displayed as buttons at the right side of the page. They are divided into three groups: “Mapping” tags include metadata fields that have equivalent meaning, content, and description in a data or metadata source, e.g. NCBI Virus [11]; “Taxonomy” tags include fields that are part of a public taxonomic ontology, e.g. ICTV Taxonomy [17]; and “Semantic” tags include fields that are related according to a common topic, e.g. Host. Toggling a Tag button in the Metadata Explorer will show all metadata fields associated with this tag in the box “Selected Fields” on the left. Hovering over a field will show additional information, including a description, examples and the full list of tags. Selected fields and their descriptive information can be downloaded in XLSX, JSON, and CSV format via the buttons at the bottom. Suggestions for additions and improvements to the metadata schema can be made through the dedicated metadata GitHub repository (https://github.com/VirJenDB/virjendb_metadata).

### Application Programming Interface (API) and SwaggerUI

For advanced users, the full dataset and metadata are available through programmatic requests via a RESTful API as specified in an OpenAPI definition and accessible via SwaggerUI (Fig. 3E). The documentation page contains instructions on how to use the SwaggerUI (https://doc.virjendb.org). The Swagger UI at (https://api.virjendb.org/swagger) comprises three sections: Search, Download, and Metadata, each offering two API endpoints. Most of the requests are implemented as POST and require JSON input. The Search section accepts a JSON body containing multiple search terms combined with logical operators, with additional filtering, sorting, and constraint options. The Download section enables downloading a list of metadata records with sequences in multiple formats. Last, the Metadata section returns metadata schema information, filterable by tags, and available in multiple formats.

### Ongoing assessments of community needs

Since 2021, the needs of the virology, virus bioinformatics, and phage ecology research communities have been assessed leveraging mainly the EVBC network [22, 24, 38]. This included regular surveys at the yearly Virus Bioinformatics conference, other meetings including the German Society for Virology and personal interviews with experts and developers. Table 3 lists the most important needs of the community for a virus database as collected by the VirJenDB team, as well as the extent to which they have been addressed in the current release or are slated for future releases based on self-assessment.

**Table 3.** List of agglomerated community needs surveyed from the virus research community by the VirJenDB with their degree of implementation

Category	Community need	VirJenDB features	Degree of implementation
General	User friendliness	Web portal	+++
	Open and FAIR	Web portal	++
	Broad availability of viruses	Datasets	+++
	Workbench	Workbench	+
	Useful search (important results first)	Search	++
	Taxonomy Browsing	Datasets and Browse	++
Metadata	Taxonomic Information below species level	Datasets and Search	++
	Curated Data (sequence quality and metadata correctness)	Datasets	++
	Epidemiological metadata (Collection Date, Collection Country, Collection Coordinates	Metadata	++
Annotations	RNA secondary and tertiary structures	Cross-linking in metadata	+
	miRNA-binding sites	Metadata	+
	Host reaction/signaling pathways	Metadata	+
Webtools and analyses	Sequence search, comparisons	Tools	+
	Alignments & Phylogenetic Analysis	Tools, Datasets	+
Data	Microscopy images	Cross-linking in metadata	+
	Data upload	User-contributed metadata and datasets	+

+++ fully implemented

++ partially implemented

+ planned

## Discussion

### Dataset and resources

The full VirJenDB dataset allowed us to obtain statistics for a snapshot of current knowledge on viruses, based directly on the sequences and metadata pulled from 16 sources (Tables 1 and 2). The full dataset of 15.4 million sequences reflected strong host biases, most likely characteristic of current sequencing efforts and research priorities which target human viruses. This is visible in the vOTU summary visualization (Supplementary Fig. 1B) of the 0.9 million vOTUs, of which ∼175 000 including the 8 largest clusters were SARS-CoV-2, representing 8.1 million sequences. Visualization of the dataset enabled us to explore the effects of curation as well as indicate areas for improvement. For example, the 100 connected components in the UMAP plot (Fig. 2), revealed a large “hairball” of SARS-CoV-2 genomes, which upon closer inspection were all created from a tiled amplicon panel and filled in with N’s. The same analysis also revealed the unexpected grouping of sequences from Rotavirus A and SARS-CoV-2 as well as Influenza A and HBV. This mismatch illustrates ongoing challenges in interpreting viral sequence data, as RNA viruses and eukaryotic viruses, the largest groups in VirJenDB, remain under-represented in the CheckV database [26]. Additionally, proper handling of segmented viruses requires further development. VirJenDB currently stores individual genome segments as separate records. In future updates, we will implement an additional metadata field to link segments belonging to the same virus genome, enabling straightforward extraction and analysis of complete segmented viral genomes as coherent units.

From the construction of the dataset and portal, which was done with regard to Open Science and FAIR principles as described in Table 4 and requested by the community as listed in Table 3, we provide several versioned resources which are freely available at the Datasets page. The VirJenDB datasets are intended to be resources for researchers for use in meta and other analyses. One interesting application may be the use of the vOTU cluster dataset in metagenomics analyses as a reference dataset as sequences from the entire virus taxonomy are included together with curated environmental metadata. Our virus–host mappings form a resource for exploring host range, with multiple available outputs including our metadata schema, source mappings, and sequence datasets. Further directions of the dataset construction include adding protein sequences, metagenome and other -omics layers, and links to protein structures and microscopy datasets.

**Table 4.** FAIR principles implementation regarding the VirJenDB records

	**Findable**
F1*	Each virus record has a unique VirJenDB Accession which allows it to be searched on the portal. These and subsets are planned to be registered as DOIs in a future release.
F2*	The virus records contain rich metadata structured within the VirJenDB v1.0 metadata schema fields. Further enrichment is planned through curation.
F3	The metadata clearly and explicitly includes the VirJenDB Accession, allowing linkage to the sequence for each record.
F4*	Each virus record is indexed in the VirJenDB search engine. Future plans include a registration service and integration into larger infrastructures, e.g. the NFDI4Microbiota central web portal and the CLoWM workflow management system.
	**Accessible**
A1.1	Each virus record is retrievable by its VirJenDB Accession through the search and API on the publicly accessible web portal.
A1.2	No authentication or authorization procedure is necessary to retrieve a VirJenDB virus record as these are publicly available through the web portal.
A2*	For each virus record, a limited set of metadata is planned to be available on Aruna, enabling the accessibility of the metadata even if the virus sequence is not available.
	**Interoperable**
I1*	The virus records and metadata are currently available in tabular (TSV, CSV, and XLSX) and JSON formats, which are formal, accessible, shareable, and broadly applicable. Formalization into a language such as SPARQL or LINKML is planned.
I2*	The VirJenDB uses data sources that use the MiXS standards, which apply controlled vocabularies that follow FAIR principles. (e.g. ENV-O: http://www.obofoundry.org/ontology/envo.html, NCBITAXON: http://purl.bioontology.org/ontology/NCBITAXON/131567). Further and ongoing integration with FAIR ontologies are planned.
I3	The virus records and metadata contain identifiers enabling cross-linking to data sources and other resources, comprising qualified references to other (meta)data.
	**Reusable**
R1*	The virus records are richly described with a plurality of accurate and relevant attributes, including 10 derived fields, and are planned to be maintained and regularly improved.
R1.1	The use of the VJDB portal, code, tools, and data, unless otherwise noted, is available under a CC-BY license as stated on the web portal and documentation.
R1.2	The virus records are associated with detailed provenance listed in the full metadata schema and in this manuscript.
R1.3	The sequences of each virus record are accessible in domain-relevant community standard formats (FASTA, GZ).

We evaluated the FAIR aspects of the VirJenDB virus records according to the FAIRification Framework (https://www.go-fair.org/fair-principles). Asterisks * indicate if the FAIR aspect is planned or under development at the time of publication.

Regular updates, curation, maintenance, and improvements of the VirJenDB dataset are planned. Three layers of data backup are implemented to ensure reliability and long-term preservation. Specifically, the system performs automatic daily incremental snapshots directly from the Elasticsearch indices, creating a continuous record of all changes. In addition, once a month we generate a complete JSON dump of the entire database, which is stored in a secure, redundant volume. Finally, every 3 months, we perform a full snapshot of both the virtual machines and storage volumes, thereby ensuring disaster recovery at the infrastructure level.

We plan to implement a rolling update strategy. Rolling updates are essential because the sources integrated into VirJenDB, such as NCBI GenBank, are continuously updated. The update strategy is two-tiered: the current strategy comprises manual data ingestion, together with semi-automatic curation scripts and data integration. These updates occur roughly every 3 months; automated pipelines for data ingestion are being constructed. These pipelines will include tracking of updates from the data sources via available APIs, programmatic or web-accessible downloads, for example using the relatively new NCBI Datasets feature (https://www.ncbi.nlm.nih.gov/datasets). Once the automatic pipelines are operational, daily minor dataset releases are planned, with major releases featuring new data sources, web portal features occurring less frequently, using “version major.minor” incrementation. Manual mapping of new and updated metadata sources are planned to continue in addition to community curation and schema development. This approach can ensure both timeliness and quality, while avoiding service interruptions and preserving continuity for users.

### Tools

While bioinformatics tools are continually modernized to process the increasing volumes of omics data, the implementation of these tools by primary repositories and analysis platforms can take time. VirJenDB serves as a flexible testing ground for tool developers, both by providing access to a large, versioned dataset of sequences and curated metadata, and by making the downstream data layers accessible to the community. For example, we applied the recently published fast virus sequence clustering tool Vclust on all VirJenDB sequences and integrated the results into the portal within approximately two working months [36]. As a virus node within the NFDI4Microbiota consortium, we help recruit, embed, and integrate virus bioinformatics tools from the community to provide additional relevant metadata fields and derived data layers to share with other databases and researchers. Tool integration was requested by the community in the last few years, which we plan to implement as listed in Table 3. Requests for specific tools and for collaboration are welcome through the channels featured on our Contact Us page (https://virjendb.org/ContactUs).

### User-contributed metadata and datasets

While the full dataset was constructed with attention to data quality, it is important to realize that no curation is ever complete, as it remains an ongoing effort. Notably, some host annotations are spurious, especially for viruses of prokaryotes, as exemplified by the human host annotations of some of the common human gut-associated bacteriophage crAssphage [37]. Further, solutions for different challenges are still needed by the eukaryotic virus community [38]. We welcome curation contributions from the community leveraging our soon to be released Github subrepository for curation. In the meantime, interested parties can contact us through the channels featured on our Contact Us page: email, feedback form, and GitHub pull requests and issues (https://virjendb.org/ContactUs).

Efforts to curate virus metadata often occur at the level of individual research projects, with the resulting linked, structured, and cleaned metadata deposited into generalized repositories. These present useful but scattered sources of curated virus metadata (see Supplementary Table S2 for examples). Here, we provide a centralized place to integrate structured sources of virus metadata that would otherwise remain distributed and may become outdated. To contribute an already existing resource of virus metadata, sequences or derived information from sequences, we ask the community to send us their publications and linked datasets through the Contact Us page. Through the VirJenDB portal and infrastructure, we aim to serve as a central hub for curation efforts across diverse viral subdomains, with the final goal of helping to develop ontologies and correcting the data in the source, i.e. user-submissions in GenBank and ENA. This includes supporting the development of expanded metadata schemas to enhance virus dataset submissions to INSDC, leveraging the database’s training resources, support services, and networking opportunities.

### Community feedback mechanisms

**Figure 4.**
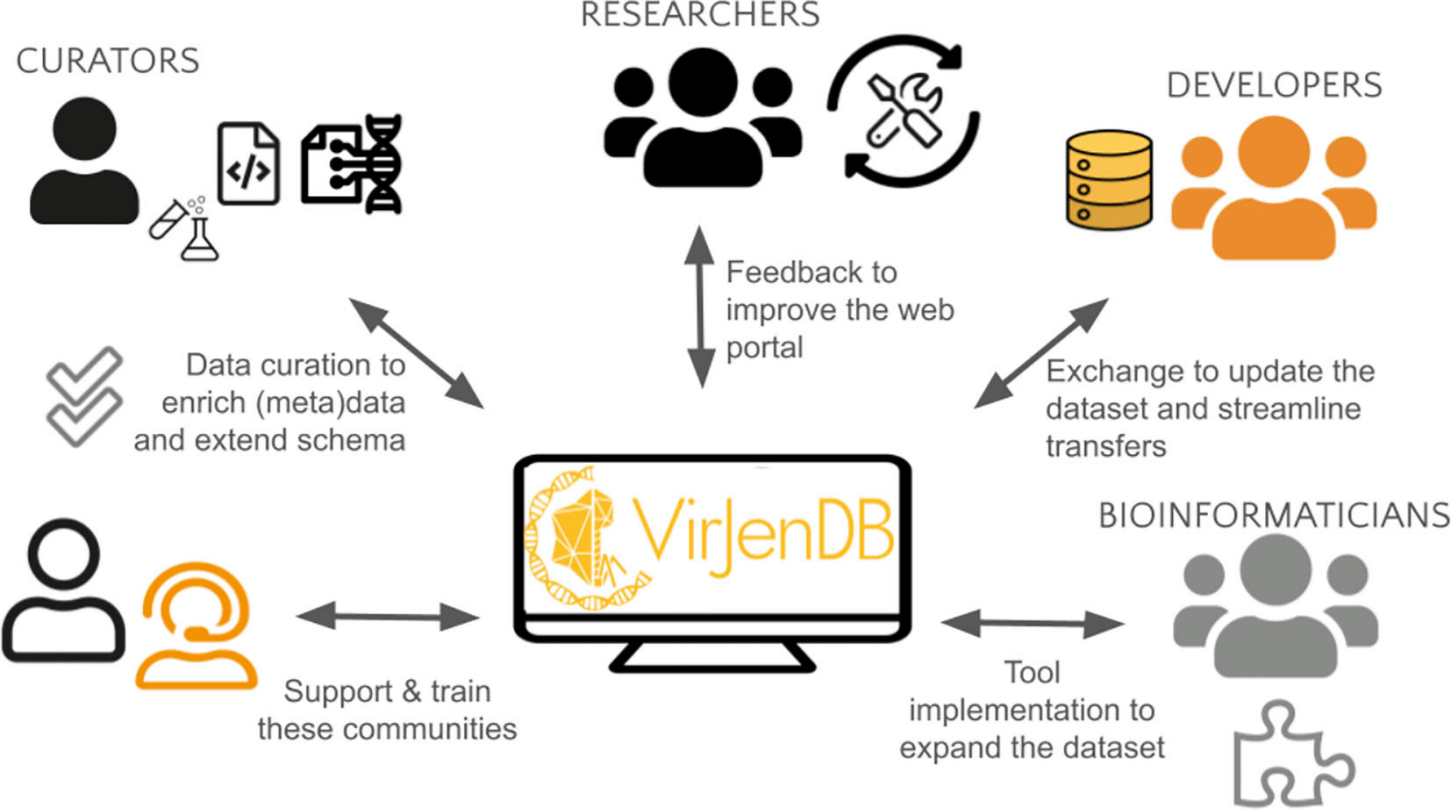
Community engagement aspects for improving VirJenDB. Regular improvement of the portal, dataset, and metadata is carried out based on feedback from users, including communities of curators, researchers, infrastructure developers, and bioinformaticians.

The community feedback mechanisms currently include presentation and demos of the VirJenDB at workshops and conferences (Fig. 4), and one-on-one longer meetings with virus and bioinformatics experts [24]. We collect feedback regarding the functionality of the portal and add to the workstream, for example the previous needs which can be found in Table 3. Other sources of continuous feedback include a form on the website (Contact Us page, Fig. 3A) and issues and pull requests on the VirJenDB GitHub repository (https://github.com/VirJenDB). In the future, these mechanisms will expand to incorporate tools, pipelines, and workflows contributed by users, as well as processes for data submission and correction to the original repository.

### Summary

The VirJenDB serves as a resource to bridge gaps between the phage and eukaryotic virus research communities, both nationally and internationally. Leveraging the VirJenDB dataset, meta-analyses will allow researchers to uncover novel patterns, relationships, and trends across virus-related research domains such as epidemiology, agriculture, biodiversity, evolution, and ecology. The open, modular development approach enables the integration of bioinformatics tools, the sharing of flexible data layers, and serves as a long-term strategy in case of uncertain future funding. By facilitating the (re)use of viral sequences and metadata in adherence to FAIR (Findable, Accessible, Interoperable, and Reusable) and Open Science principles, the VirJenDB fosters greater transparency, accessibility, and ultimately, trust in the global virus research infrastructure.

## Supplementary Material

gkaf1224_Supplemental_File

## Data Availability

VirJenDB is available at https://www.virjendb.org.
