# Supplementary material for: VirJenDB: a FAIR (meta)data and bioinformatics platform for all viruses
Source: Nucleic Acids Res. 2025 Dec 17;54(D1):D912–24. doi: 10.1093/nar/gkaf1224 (PMC12807664; doi:10.1093/nar/gkaf1224)
Supplement: gkaf1224_Supplemental_File [file gkaf1224_supplemental_file.pdf]

VirJenDB: a FAIR (meta)data and analysis platform for all viruses

Shahram Saghaei, Malte Siemers, Kilian L. Ossetek, Stephan Richter, Robert A. Edwards, Simon Roux, Andrzej Zielezinski, Bas E. Dutilh, Manja Marz, and Noriko A. Cassman

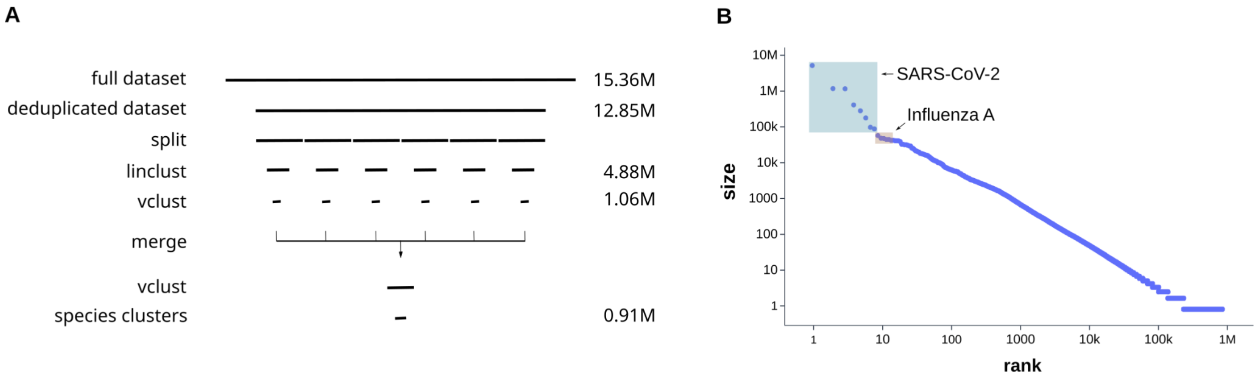

Supplementary Figure 1. Diagram of the VirJenDB clustering workflow. A) Number of sequences after each step in the clustering workflow with the full dataset at the top going down to the approximate species-rank vOTU cluster representative dataset at the bottom. B) Distribution of the sizes encompassing the sorted number of sequences within each cluster. The 8 largest clusters on the left represent the largest clusters, comprising sequences from SARS-CoV-2 (grey), and the following 11 clusters are identified as Influenza A virus (orange). At the right, 600 thousand singleton clusters are shown.

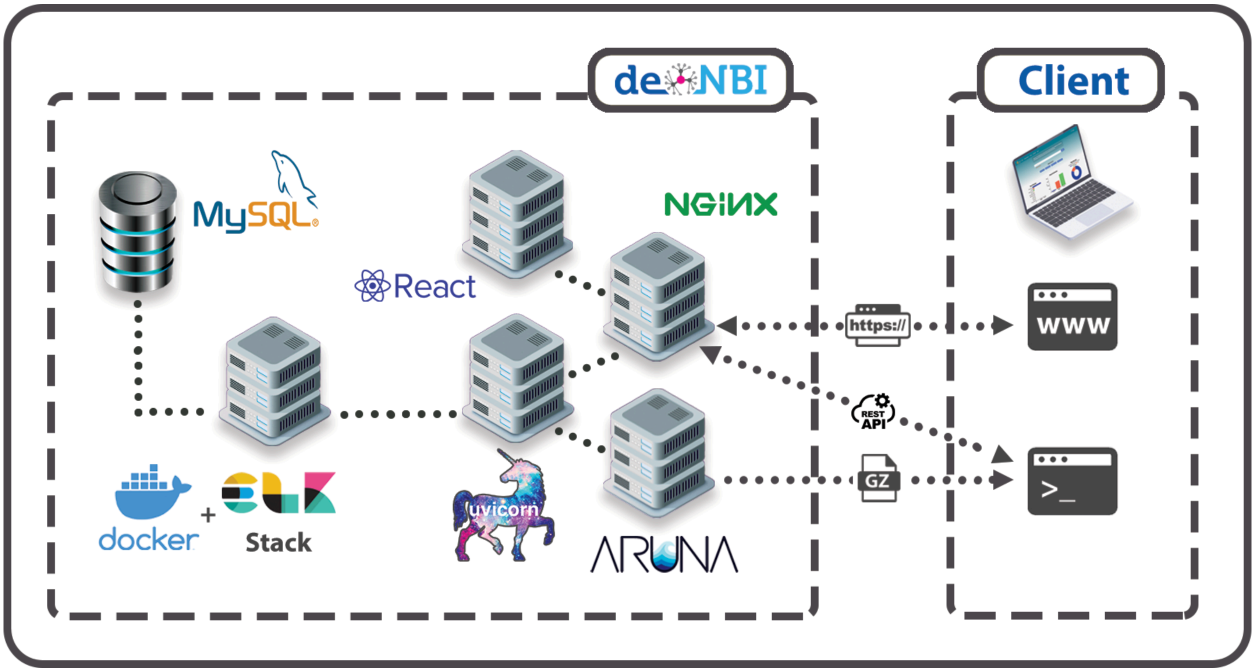

Supplementary Figure 2. Network diagram of the backend VirJenDB components.
The backend architecture is hosted on the de.NBI infrastructure and uses Docker containers for deploying some of the services. The ELK Stack (Elasticsearch, Logstash, and Kibana) serves as the primary database and query engine, enabling efficient data indexing and search. MySQL is used as a backup database to ensure data persistence and reliability. ARUNA functions as the storage layer for data management and retrieval. The backend services, running on Uvicorn (a Python-based API server), handle client requests through a REST API. The React frontend is served via NGINX, which manages HTTPS connections and routes requests to the backend. Clients interact with the system either through a web browser or command-line tools using secure HTTPS and REST API endpoints. This architecture provides a scalable, maintainable, and secure environment for data processing and user access.

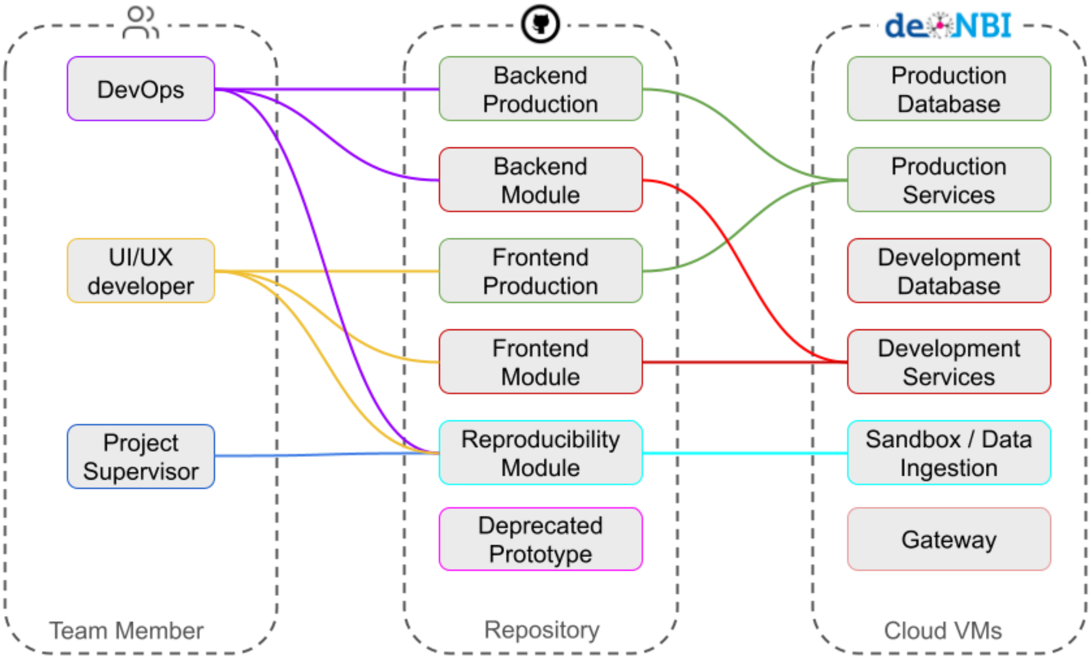

Supplementary Figure 3. Development stream facilitated through the VirJenDB GitHub repository. The diagram shows the system architecture and interactions between its components. Dashed boxes represent different domains or roles: the team member domain includes users and roles interacting with the system, the repository domain represents the code components, and the cloud virtual machine domain corresponds to the system services and infrastructure. Solid boxes indicate individual components within each domain, illustrating the relationships between users, code, and infrastructure. The Development and Operations engineer interacts with backend repositories, while User Interface and Experience developer interacts with frontend repositories.

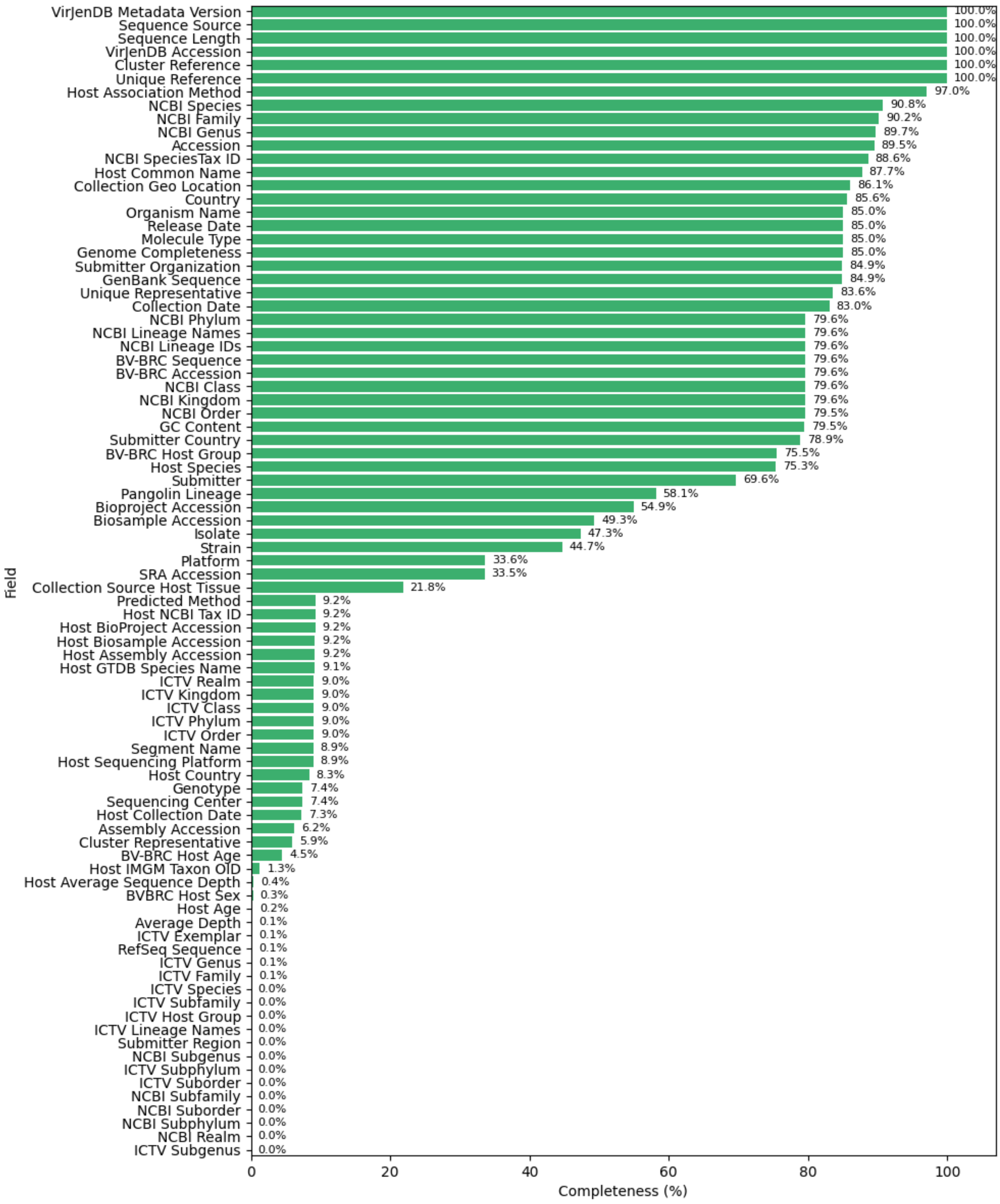

Supplementary Figure 4. Metadata field completeness abundance plot of the VirJenDB full dataset including the 76 non-empty public fields, sorted according to completeness.

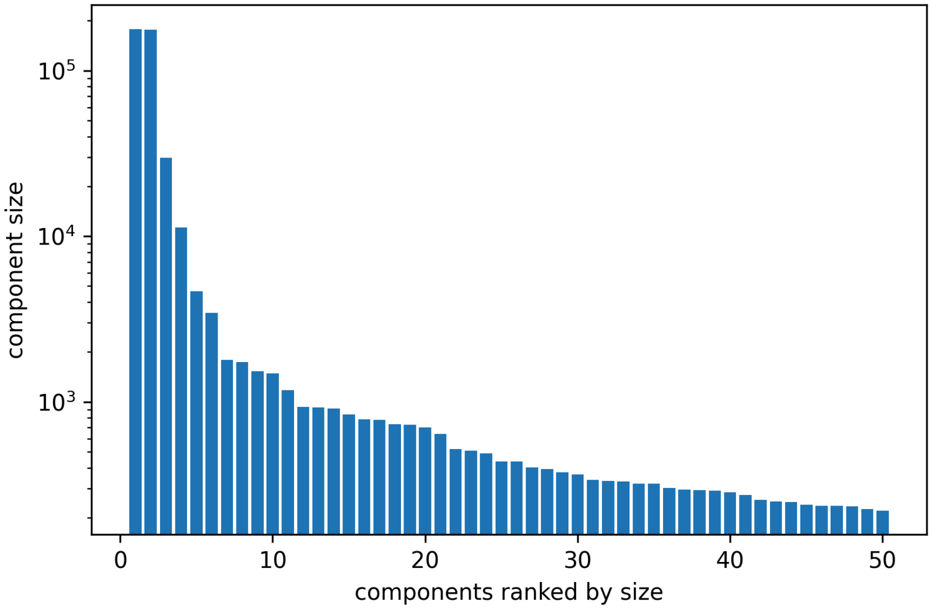

Supplementary Figure 5. Sizes (in log scale) of the 50 largest connected components in the clustering of the approximately species-level vOTU cluster representatives for the UMAP visualization (Figure 2). The largest connected component included 175 thousand vOTU cluster representatives.

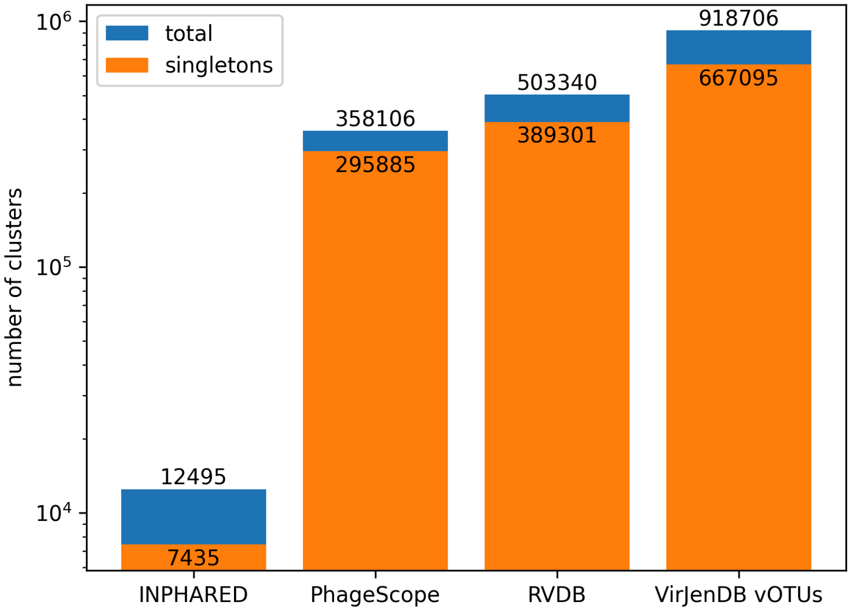

Supplementary Figure 6. Barplot comparison of the number of clusters in INPHARED, PhageScope, RVDB and VirJenDB. All datasets were clustered with Vclust v1.30 at 95% ANI over 85% length with the Leiden algorithm. Singletons refer to clusters with only a single sequence.

Supplementary Table 1. Abbreviated version of the VirJenDB v1.0 metadata schema. The full schema is available in multiple formats on the Metadata Explorer (https://virjendb.org/MetadataExplorer) and in the Documentation (https://doc.virjendb.org/pages/metadata.html).

Field ID	Name	Description	Input Source	Example
insdc_accession_id	Accession	GenBank Accession of the virus sequence. It equals NCBI Accession, ENA Accession, and DDBJ Accession, which are part of the INSDC.	NCBI Virus|BVBRC	
assembly_accession_id	Assembly Accession	NCBI Assembly Accession of the virus sequence.	NCBI Virus|BVBRC	
bioproject_accession_id	Bioproject Accession	References the BioProject Accession that the virus sequence is a part of (INSDC).	NCBI Virus|BVBRC	
biosample_accession_id	Biosample Accession	References the BioSample Accession that the virus sequence is a part of (INSDC).	NCBI Virus|BVBRC	
collection_date	Collection Date	Date when the sample the virus sequence originates from was collected or sampled.	NCBI Virus|BVBRC	2023-05-28
collection_country	Country	Country or sea area where the sample the virus sequence originates from was collected or sampled.	NCBI Virus|BVBRC	Germany
is_genbank_sequence	GenBank Sequence	Determines if this virus sequence is available in Genbank.	NCBI Virus	true
is_refseq_sequence	RefSeq Sequence	Determines if this virus sequence is a reference sequence in the RefSeq database.	NCBI Virus|BVBRC	true
collection_geo_location	Collection Geo Location	Country name and additional identifier like a state abbreviation from where the sample the sequence originates from was collected/sampled.	NCBI Virus|BVBRC	USA: NY

host_natural_common_name	Host Common Name	Common name of the host the virus sequence was collected from, e.g. human. Currently (v1.0) no ontology is applied.	NCBI Virus|BVBRC	human
sequence_length	Sequence Length	Length of the virus sequence in basepairs (bp).	NCBI Virus|BVBRC	10201
molecule_type	Molecule Type	Molecule type of the sequence, e.g. ssRNA. Note that there are 15 molecule types corresponding to combinations of the Baltimore Classifications, from NCBI GenBank.	NCBI Virus	ssRNA(+)
sequence_completeness	Genome Completeness	Whether the sequence is a partial or complete sequence in NCBI GenBank, "Nuc_Completeness" from NCBI Virus	NCBI Virus	partial; complete
organism_name	Organism Name	NCBI GenBank organism name: a taxonomic name at species level or below the species level.	NCBI Virus	Influenza A virus (A/goose/Guangdong/1/1996(H5N1))
submitter_organization	Submitter Organization	Organization the submitter(s) is(are) affiliated with.	NCBI Virus	National Center for Biotechnology Information, NIH
sequence_repository_release_date	Release Date	Date when the sequence was made public in a repository (e.g. GenBank, BV-BRC).	NCBI Virus	2023-05-20
sequence_segment_name	Segment Name	Name of the virus segment. Can be a number.	NCBI Virus	spike; 1; whole genome
sra_accession_id	SRA Accession	NCBI Sequence Read Archive (SRA) Accession.	NCBI Virus|BVBRC	
submitter	Submitter	Name(s) of the submitter(s) of the virus sequence.	NCBI Virus	Zuckerman,N., Mandelboim,M.
collection_source_host_tissue	Collection Source	Tissue, Specimen or Source of the superhost from which the sample	NCBI Virus	brain; water; venom gland

	Host Tissue	the virus sequence originates from was collected or sampled.		
bvbrc_accession_id	BV-BRC Accession	Accession of the sequence in the BV-BRC database.	BVBRC	
host_group	BV-BRC Host Group	The host's broader group association in a taxonomic context from BV-BRC.	BVBRC	vertebrates; plants
host_natural_scientific_name	Host Species	The species name of the host associated with the virus sequence.	BVBRC	homo sapiens
host_ncbi_tax_id	Host NCBI Tax ID	The host sequence NCBI Taxonomy ID. Can be based on the mapping by GTDB of the GTDB Species Name to the NCBI Taxonomy ID.	GTDB	9606
host_sex	BVBRC Host Sex	Sex of the associated host from the BV-BRC.	BVBRC	female; hermaphrodite
is_bvbrc	BV-BRC Sequence	Flag indicating if this virus sequence is available in BV-BRC.	VJDB	true
pangolin_lineage	Pangolin Lineage	Lineage determined by Pangolin.	NCBI Virus|BVBRC	
sample_ncbi_tax_id	NCBI SpeciesTax ID	The virus species NCBI Taxonomy ID.	BVBRC	197911
sequence_average_depth_coverage	Average Depth	Average sequencing depth across the sequence from BVBRC.	BVBRC	12.0x
sequence_gc_content	GC Content	Percentage of G and C nucleotides in the sequence from BVBRC.	BVBRC	37.355248
sequencing_center	Sequencing Center	Institution that sequenced the collected sample.	BVBRC	US Food and Drug Administration

sequencing_platform	Platform	Instrument platform used for sequencing the virus; multiple values are seperated by a semicolon (only for assemblies).	BVBRC	CAPILLARY
source_metadata_db	Sequence Source	The name of the source database or tool by which the virus sequence was added in VirJenDB; in the case of PhiSpy-predicted prophages, the value is PhiSpy.	VJDB	
submitter_country	Submitter Country	Country of the submitter's organization.	NCBI Virus	Germany
submitter_region	Submitter Region	Region or location of the organization of the submitters.	NCBI Virus	
virjen_id	VirJenDB Accession	Unique identifier of the virus sequence, assigned by VirJenDB.	VJDB	10580360
virjen_version	VirJenDB Metadata Version	Version of the virus sequence record, including metadata, assigned by VirJenDB.	VJDB	
strain	Strain	Name of the virus strain associated with the sample from which the virus sequence was obtained.	BVBRC	
ictv_realm	ICTV Realm	A 'Realm' is the highest taxonomic rank into which virus species can be classified. Defined by the ICTV.	ICTV	
ictv_kingdom	ICTV Kingdom	A 'Kingdom' is a rank in the taxonomic hierarchy into which virus species can be classified. Defined by the ICTV.	ICTV	
ictv_phylum	ICTV Phylum	A 'Phylum' is a rank in the taxonomic hierarchy into which virus species can be classified. Defined by the ICTV.	ICTV	
ictv_subphylum	ICTV Subphylum	A 'Subphylum' is a rank in the taxonomic hierarchy into which virus species can be classified. Defined by the ICTV.	ICTV	

ictv_class	ICTV Class	A 'Class' is a rank in the taxonomic hierarchy into which virus species can be classified. Defined by the ICTV.	ICTV	
ictv_order	ICTV Order	A 'Order' is a rank in the taxonomic hierarchy into which virus species can be classified. Defined by the ICTV.	ICTV	
ictv_suborder	ICTV Suborder	A 'suborder' is a rank in the taxonomic hierarchy into which virus species can be classified. Defined by the ICTV.	ICTV	
ictv_family	ICTV Family	A 'Family' is a rank in the taxonomic hierarchy into which virus species can be classified. Defined by the ICTV.	ICTV	
ictv_subfamily	ICTV Subfamily	A 'Subfamily' is a rank in the taxonomic hierarchy into which virus species can be classified. Defined by the ICTV.	ICTV	
ictv_genus	ICTV Genus	A 'Genus' is a rank in the taxonomic hierarchy into which virus species can be classified. Defined by the ICTV.	ICTV	
ictv_subgenus	ICTV Subgenus	A 'Subgenus' is a rank in the taxonomic hierarchy into which virus species can be classified. Defined by the ICTV.	ICTV	
ictv_species	ICTV Species	A Species is the lowest taxonomic rank in the hierarchy approved by the ICTV. While subspecies levels of classification may exist for some viruses (e.g. Hepatitis C virus), the ICTV does not classify viruses below the species level. Defined by the ICTV.	ICTV	

ncbi_realm	NCBI Realm	A 'Realm' is the highest taxonomic rank into which virus species can be classified. Defined by the NCBI.	NCBI Taxonomy	
ncbi_kingdom	NCBI Kingdom	A 'Kingdom' is a rank in the taxonomic hierarchy into which virus species can be classified. Defined by the NCBI.	BVBRC	
ncbi_phylum	NCBI Phylum	A 'Phylum' is a rank in the taxonomic hierarchy into which virus species can be classified. Defined by the NCBI.	BVBRC	
ncbi_subphylum	NCBI Subphylum	A 'Subphylum' is a rank in the taxonomic hierarchy into which virus species can be classified. Defined by the NCBI.	NCBI Taxonomy	
ncbi_class	NCBI Class	A 'Class' is a rank in the taxonomic hierarchy into which virus species can be classified. Defined by the NCBI.	BVBRC	
ncbi_order	NCBI Order	A 'Order' is a rank in the taxonomic hierarchy into which virus species can be classified. Defined by the NCBI.	BVBRC	
ncbi_suborder	NCBI Suborder	A 'suborder' is a rank in the taxonomic hierarchy into which virus species can be classified. Defined by the NCBI.	NCBI Taxonomy	
ncbi_family	NCBI Family	A 'Family' is a rank in the taxonomic hierarchy into which virus species can be classified. Defined by the NCBI.	NCBI Virus|BVBRC	
ncbi_subfamily	NCBI Subfamily	A 'Subfamily' is a rank in the taxonomic hierarchy into which virus species can be classified. Defined by the NCBI.	NCBI Taxonomy	

ncbi_genus	NCBI Genus	A 'Genus' is a rank in the taxonomic hierarchy into which virus species can be classified. Defined by the NCBI.	NCBI Virus|BVBRC	
ncbi_subgenus	NCBI Subgenus	A 'Subgenus' is a rank in the taxonomic hierarchy into which virus species can be classified. Defined by the NCBI.	NCBI	
ncbi_species	NCBI Species	A Species is the lowest taxonomic rank in the hierarchy approved by the NCBI. While subspecies levels of classification may exist for some viruses (e.g. Hepatitis C virus), the NCBI does not classify viruses below the species level. Defined by the NCBI.	NCBI Virus|BVBRC	
virus_genotype	Genotype	The genotype or subtype of a virus sequence, as provided by the sequence submitter. This field comes from the "/serotype" field of the GenBank record and is shown as submitted. Consistency and accuracy may vary.	NCBI Virus	
virus_isolate	Isolate	Name of the virus isolate associated with the sample from which the virus sequence was obtained.	NCBI Virus	
host_age	Host Age	Host age number only from "BVBRC Host Age". Caution: not yet standardized by unit!	BVBRC	37 years
ncbi_taxon_lineage_ids	NCBI Lineage IDs	NCBI TaxIDs assigned to the virus sequence, from BVBRC.	BVBRC	

ncbi_taxon_lineage_names	NCBI Lineage Names	Aggregation of the NCBI Taxonomy Names associated with the virus sequence.	BVBRC	
ictv_taxon_lineage_names	ICTV Lineage Names	Aggregation of the ICTV Taxonomy Names associated with the virus sequence.	VJDB	
is_ictv_exemplar	ICTV Exemplar	Flag indicating yes if the sequence is an exemplar of the species in the ICTV Taxonomy.	ICTV	
ictv_host_group	ICTV Host Group	The ICTV Host source field content to be combined with other host group data from the other sources.	ICTV	
bvbrc_host_age	BV-BRC Host Age	Host Age and Unit from BV-BRC.	BVBRC	
host_gtdb_species	Host GTDB Species Name	References the Host GTDB Species Name to which the NCBI host taxonomy ID of the virus sequence could be mapped. See the mapping file on the Datasets page.	IMG/VR|PhiSpy|PhD	
is_unique	Unique Representative	Flag indicating yes if the sequence is a representative for a group of identical VirJenDB sequences.	VJDB	
in_unique	Unique Reference	The VirJenDB ID for the unique representative sequence. For the non-phages, the smallest Accession in the group. See documentation for selection details.	VJDB	
is_cluster	Cluster Representative	Flag indicating yes if the sequence is a representative sequence of a group of sequences, computed by VClust. See documentation for selection details.	VJDB	
in_cluster	Cluster Reference	The VirJenDB ID of the representative of its cluster. See documentation for selection details.	VJDB	

predicted_method	Predicted Method	The tool used to predict the virus sequence. No value means that it is not a predicted virus sequence.	IMG/VR|PhiSpy|PhD	
host_insdc_accession_id	Host Accession	Host Accession from NCBI GenBank that refers to the host sequence from which the prophage was predicted.	VJDB	
host_assembly_accession_id	Host Assembly Accession	Host Assembly Accession of the sequence (INSDC).	IMG/VR|PhiSpy|PhD	
host_bioproject_accession_id	Host BioProject Accession	References the BioProject Accession associated with the host sequence.	IMG/VR|PhiSpy|PhD	
host_biosample_accession_id	Host Biosample Accession	References the BioSample Accession associated with the host sequence.	IMG/VR|PhiSpy|PhD	
host_collection_country	Host Country	Country or sea area origin of the sample from which the host sequence originates.	IMG/VR|PhiSpy|PhD	Germany
host_collection_date	Host Collection Date	Date of collection of the sample from which the host sequence originates.	IMG/VR|PhiSpy|PhD	2023-05-23
host_imgvr_taxon_oid	Host IMGM Taxon OID	Associated Host IMGM Taxon OID from which the virus sequence was extracted by IMG/VR.	IMG/VR|PhiSpy|PhD	
host_sequence_average_depth_coverage	Host Average Sequence Depth	Average sequencing depth across the host sequence. Multiple host associations/predictions separated by semicolons.	IMG/VR|PhiSpy|PhD	12.0x
host_sequencing_platform	Host Sequencing Platform	Instrument or sequencing platform used for host sequencing. Multiple host associations/predictions separated by semicolons.	IMG/VR|PhiSpy|PhD	CAPILLARY
host_association_method	Host Association Method	Method by which the fields "Host Accession ID" from NCBI GenBank or "NCBI Taxonomy Lineage" was associated to the virus sequence. Currently (v1.0) either a host association from the	VJDB	

		data source or PhiSpy prophage prediction. Multiple host associations/predictions separated by semicolons.		

Supplementary Table 2. List of virus metadata and genome sources deposited into unstructured repositories.

Source Type	URI
genome catalog	https://www.cell.com/cell/fulltext/S0092-8674(19)30341-1
metadata	https://figshare.com/articles/dataset/A_DATASET_OF_DISTRIBUTION_AND_DIVERSITY_OF_MOSQUITO-ASSOCIATED_VIRUSES_AND_THEIR_RELATED_MOSQUITO_VECTORS_IN_CHINA/12638792
metadata	https://www.sciencedirect.com/science/article/pii/S2352771423000101#ec0010
genome catalog	http://onlinelibrary.wiley.com/doi/10.1111/j.1462-2920.2012.02891.x/abstract
genome catalog	http://www.nature.com/articles/nature19094
genome catalog	https://www.nature.com/articles/ismej2017157
genome catalog	https://www.nature.com/articles/ncomms15955
genome catalog	http://journal.frontiersin.org/article/10.3389/fmicb.2017.00352/full
genome catalog	https://www.cell.com/cell-reports/fulltext/S2211-1247(21)00471-X
